# Is there a dysfunction in the visual system of depressed patients?

**DOI:** 10.1186/1744-859X-4-7

**Published:** 2005-03-29

**Authors:** Konstantinos N Fountoulakis, Fotis Fotiou, Apostolos Iacovides, George Kaprinis

**Affiliations:** 1 Laboratory of Psychophysiology, 3^rd ^Department of Psychiatry, Aristotle University of Thesssaloniki, Greece; 2 Laboratory of Clinical Neurophysiology, 1^st ^Department of Neurology, Aristotle University of Thesssaloniki, Greece

**Keywords:** EOG, ERG, depression, Visual system.

## Abstract

**Background:**

The aim of the current study was to identify a possible locus of dysfunction in the visual system of depressed patients.

**Materials and Methods:**

Fifty Major Depressive patients aged 21–60 years and 15 age-matched controls took part in the study The diagnosis was obtained with the SCAN v 2.0. The psychometric assessment included the HDRS, the HAS, the Newcastle Scales, the Diagnostic Melancholia Scale and the GAF scale. Flash Electroretinogram and Electrooculogram were performed in all subjects. The statistical analysis included ANCOVA, Student's t-test and Pearson Product Moment Correlation Coefficient were used.

**Results:**

The Electro-oculographic findings suggested that all subtypes of depressed patients had lower dark trough and light peak values in comparison to controls (p < 0.001), while Arden ratios were within normal range. Electroretinographic recordings did not reveal any differences between patients and controls or between subtypes of depression.

**Discussion:**

The findings of the current study provide empirical data in order to assist in the understanding of the international literature and to explain the mechanism of action of therapies like sleep deprivation and light therapy.

## Background

Depression, according to recent epidemiological surveys might affect almost 25% of the general population at some point of their lives. The definition of 'depression' according to both classification systems [1-3], is based on the definition of the depressive episode. Modern classification systems recognise melancholic ('somatic) and atypical features. In spite of early reports [4-7], today the only report which seems to survive is not the favourable response of atypical patients to MAOIs, but their resistance to TCAs.

One of the theories concerning the etiopathogenesis of depression suggests that a disturbance of biological rhythms is the core feature [8]. This disturbance is better studied in Seasonal Affective Disorder (SAD), which is a form of depression which responds to light therapy. It is possible that similar disturbances might be also present in non-seasonal depression, since these patients respond to sleep deprivation, especially in combination to light therapy. Additionally, there is a direct connection of the hypothalamus with the retina (retinohypothalamic tract) and some authors believe that at least 40% of brain neurones carry or process visual information [9].

A neglected area concerns the contribution of the visual system to the genesis of the circadian rhythms of the organism. Especially the direct assessment of retinal function would be valuable [10]. The suprachiasmatic nucleus is believed to be the center of the production of these rhythms. It processes information originating from the retina. Our group has already published papers on the visual system of depressives [11,12] and Alzheimer disease patients [13] using pupillometry. In a recent study of our group [14] the use of PR-VEPs revealed that there might be an underactivation of the anterior right hemisphere in melancholic depressives (anterior to the chiasm) and a hyperactivation of the same region in atypical depressives. The question which arises is whether there is a specific dysfunction at the level of the pigmentum epithelium or the retina responsible for these findings.

The present study **aimed **to investigate the outer part of the visual system of depressed patients and to provide evidence for further localization of a suggested anterior right hemisphere dysfunction in depression. Also aimed to compare the results of normal controls with those of depressed patients and to compare depressed subtypes between each other.

## Materials and methods

### Study Participants

Fifty (50) patients (15 males and 35 females) aged 21–60 years (mean = 41.0, standard deviation = 11.4) and 15 controls (4 males and 11 females) aged 20–55 years (mean 35.2, standard deviation = 9.2) suffering from Major Depression according to DSM-IV [2], and depression according to ICD-10 [15] criteria, took part in the study. All provided written informed consent. Fourteen of them fulfilled criteria for atypical features, 16 for melancholic features and 32 for somatic syndrome (according to ICD-10). Also, 9 patients did not fulfilled criteria for any specific syndrome (undifferentiated patients).

All were inpatients or outpatients of the 3^rd ^Department of Psychiatry, Aristotle University of Thessaloniki, University Hospital AHEPA, Thessaloniki Greece. They constituted the total number of patients during a two-years period that fulfilled the criteria to enter in the study. These criteria demanded that patients:

1. Be free of any medication for at least two weeks prior to the first assessment and diagnosis. In no case medication was interrupted in order to include the patient in the study.

2. Be physically healthy with normal clinical and laboratory findings, including EEG, ECG and thyroid function.

3. Opthalmological examination should be normal and patients should have normal or corrected visual acuity and went through a full ophthalmologic investigation.

4. No patient should fulfill criteria for catatonic or psychotic features or for seasonal affective disorder.

5. Also, no patient should fulfill criteria for another DSM-IV axis-I disorder, except from generalised anxiety disorder and panic disorder

6. No past history of manic or hypomanic episode.

7. Psychiatric history of no more than five distinct episodes including the present one (mean 1.16 ± 1.53).

8. Patients should be right-handed and the right eye to be the dominant one.

9. All should be born and lived in the area of Thessaloniki, Greece (Latitude 40–40.1° North).

10. All should be depressed during testing.

Finally, the study sample of the current paper is identical with that of our previous study on PR-VEPs in depression [14].

#### Clinical Diagnosis

The Schedules for Clinical Assessment in Neuropsychiatry version 2.0 (SCAN v 2.0) [16] were used for the clinical diagnosis. Each one of the symptoms (according the lists of both classification systems) was recorded and correlated with the laboratory findings.

#### Laboratory Testing

It included ECG, EEG, blood and biochemical testing, test for pregnancy, T3, T4, TSH, B12 and folic acid.

#### Psychometric Assessment

Its aim was the quantification of depression and anxiety [17,18]. This was achieved with the use of the Hamilton Depression Rating Scale (HDRS) [19,20] and the Hamilton Anxiety Scale (HAS) [21] and their subscales. The assessment of the endogeneity of depression was achieved with the use of the Newcastle Scales (1965 Newcastle Depression Diagnostic Scale-1965-NDDS and 1971 Newcastle Depression Diagnostic Scale-1971-NDDS) and the Diagnostic Melancholia Scale (DMS). These three scales have a different rational in assessing the 'endogenous-melancholic' and the 'neurotic' syndromes of depression. The General Assessment of Functioning Scale (GAF) [22] was used to assess the severity of depression. The questionnaire of Holmes [23] was used to search for stressful life events during the last 6 months before the onset of the symptomatology.

### Psychophysiological Methods

It included:

#### 1. Electro-oculogram (EOG)

which is a method with which one can study the electrical and metabolic activity of the outer layers of the retina. During the adaptation of the retina to dark, the amplitude of the EOG gradually decreases, reaching a nadir (dark trough). During the adaptation to light (ganzfeld, 1200 lux) it gradualy increases reaching a zenith (light peak). The systematic development of the method of electro-oculogram was made mainly by Arden [24,25] and the conditions for EOG recording have been coded by the International Society for Clinical Electrophysiology of Vision (ISCEV) [26] and this was kept in the current study. However some deviations from these conditions were inevitable. These included the use of 3 instead of 4 electrodes, the recording every 2 min for 12 minutes duration instead of every minute for a 15 minutes duration and not dilatated pupils.

A video camera was used to verify that the patients were following the instructions and moved eyes to catch the alternating lights.

**Figure 1:**
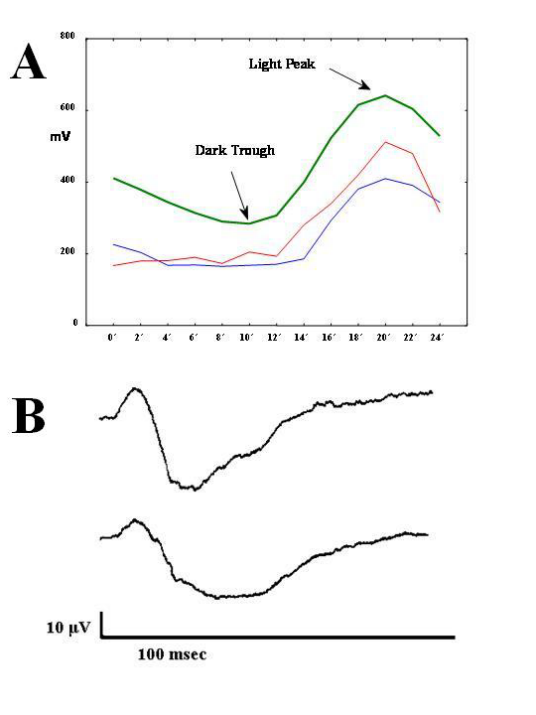
**A. Electro-oculogram (EOG)**. Recording in a normal control (upper), an atypical (middle-continuous line) and a melancholic patient (lower-dotted line). The control subject has Arden ratio = 224, the melancholic Arden ratio = 295, and the atypical patient Arden ratio = 248. However, although all ratios are within normal limits, the curves of the depressed patients have lower amplitude. **B. flash-ERG**. Upper: normal latency of a and b waves (control subject) Lower: slightly increased than normal latency of a and b waves (melancholic patient) All recordings are within normal range.

EOG was recorded by two electrodes attached in the outer canthous (Lc and Rc) and a third in the mideye (Mr). The movement of the eyes produces a change of potential, which is recorded by the electrodes. After the recording of several movements of the eyes, the averaging of potentials gives the mean potential for the given conditions (interaction of time with lighting conditions). The procedure includes recordings of eye movents every 2 minutes, for 12 minutes in dark and subsequently 12 minutes in light. The resulting recording is shown in figure 1(a).

There is no difference of the recorded EOG curves between the two eyes [27]. The most widely used indices for the interpretation of the EOG are the Arden ratio:



The normal values of this index lie between 162 and 228, but values under 180 should be considered as borderline.

Another index, which also takes into consideration the baseline potential is the A criterion [28]:

A Criterion = light peak-[0, 61*baseline potential+0,91*dark trough].

According to Pinckers over of 70% of healthy subjects have A-Criterion values over 80 and all over zero.

#### 2. Flash-Electroretinogram

This is a method of recording potentials of the retina after the fall of light stimuli. The Electroretinogram (ERG) can be recorded after flash (f-ERG) or Pattern-Reversal (PR-ERG) stimulation. In the current study, binocular f-ERG was used. ERG recording have been coded by the International Society for Clinical Electrophysiology of Vision (ISCEV) and this was kept in the current study. However some deviations from these conditions were inevitable. These included the use of skin electrodes, and lack of maximum dilatation of the pupil. The f-ERG curve includes mainly the waves a and b. Wave a is photochemical in origin and is produced in the photoreceptors as their respond to a light stimuli and under specific conditons (scotopic conditions) the a wave may be split to ap and as waves [29]. It is believed that the ap wave comes from the cones and as wave from the rods [30]. The b-wave is produced by the bioelectrical activity of the neurons of the inner grannule layer and the bipolar cells. It is neuronal in origin. It can also be split (under scotopic conditions) in two waves, named bp and bs.

In the current study, f-ERG was recorded from two electrodes, attached below the eyes (Lr and Rr) and a reference electrode at the mid-eye (Mr), under photopic conditions from both eyes simultaneously (binocular).

#### 3. Specific Issues

All recordings were conducted around mid-day (12:00 h to 16:00 h) and there was no difference in the times of the day or the season of the year the groups were studied. Gold-plated silver electrodes were used and the impedance was <4 kohms. All patients came from North Greece (Latitude 40–40.1° North).

### Statistical analysis

It included Analysis of Covariance (ANCOVA) with age as a covariate and Pearson's product moment correlation coefficient. Student's t-test was used for post-hoc comparisons.

Since 8 ANCOVAs were performed, the Bonferonni method suggests that the appropriate p-level should be <0.00625, and for practical reasons the level p < 0.005 was chosen and used also in post-hoc comparisons.

## Results

Depressed patients and controls had similar gender composition and did not differ in age (p = 0.107, table 1). Melancholic patients seemed to be marginally older (table 2). This is why age was used as covariate.

**Table 1:** Results of Electrooculographic and flash-Electroretinographic recordings of depressed patients and controls and p-values after ANCOVA with age as covariate.

	**depressed patients N = 50**		**Controls N = 15**			
	**Mean**	**S.D.**	**Mean**	**S.D.**	**P (ANCOVA)**	**P (post-hoc)**

Age	41.0	11.4	35.2	9.2	0.107	
						
**EOG results**					***0.001***	
Left Dark Trough	178.54	55.93	284.08	109.46		***0.000***
Left Light Peak	455.22	127.11	659.17	195.72		***0.000***
Right Dark Trough	169.32	54.74	283.08	96.72		***0.000***
Right Light Peak	402.40	104.13	646.58	183.92		***0.000***
Left Arden Ratio	261.04	49.67	241.88	44.81		0.227
Right Arden Ratio	248.47	53.87	233.81	34.94		0.374
Left A-Criterio	153.21	71.40	142.11	65.66		0.287
Right A-Criterio	118.40	66.41	131.85	69.12		0.534
						
**F-ERG Photopic Conditions**					0.147	
Lr a wave, ampl	4.54	1.96	4.63	1.57		
Lr a wave, lat	13.99	1.15	13.17	1.47		
Lr b wave, ampl	8.30	2.58	10.83	8.22		
Lr b wave, lat	31.85	2.85	29.53	4.62		
Rr a wave, ampl	4.51	1.75	4.30	1.71		
Rr a wave, lat	13.88	1.50	13.37	1.71		
Rr b wave, ampl	7.92	2.59	11.08	8.14		
Rr b wave, lat	31.94	2.87	29.57	4.68		

**Table 2:** Comparison between melancholic and atypical patients and controls (ANCOVA with age as covariate; significant are p-values below 0.005).

	**mean**	**s.d.**	**mean**	**s.d.**	**p**	**p**	**p**	**p**	**p**	**p**
	**Atypical features N = 14**		**Melancholic features N = 16**		**A/M ANCOVA**	**A/M Post-hoc**	**A/C ANCOVA**	**A/C Post-hoc**	**M/C ANCOVA**	**M/C Post-hoc**

Age	37.00	7.79	47.00	13.03	0.016		0.575		0.007	
Age of Onset	27.21	8.74	34.68	12.83	0.070		-		-	
Number of previous episodes	1.21	1.37	1.21	1.69	0.995		-		-	
GAF	60.36	10.65	41.16	12.10	***0.000***		-		-	
Number of PD diagnosed	0.50	0.65	0.16	0.37	0.066		-		-	
Number of PD criteria fulfilled	3.29	4.14	2.26	3.96	0.477		-		-	
Number of stressful life events	3.64	2.27	1.21	1.44	***0.001***		-		-	
HDRS-17	23.14	3.82	28.53	6.54	0.010		-		-	
										
**EOG**					0.510		***0.004***		***0.001***	
Left dark trough	187.86	47.31	181.84	49.22				0.006		***0.001***
Left light peak	488.50	132.98	443.16	124.36				0.015		***0.001***
Right dark trough	175.57	48.39	191.74	54.57				***0.001***		***0.002***
Right light peak	421.14	133.78	422.21	85.18				***0.001***		***0.000***
Left Arden ratio	262.95	47.09	246.03	40.03				0.256		0.790
Right Arden ratio	242.73	44.30	230.94	56.65				0.579		0.876
										
**Flash-ERG Photopic Conditions**					0.175		0.714		0.142	
Lr a wave, ampl	4.08	1.97	4.70	1.89						
Lr a wave, lat	13.38	1.42	14.37	0.75						
Lr b wave, ampl	7.44	2.19	8.80	1.50						
Lr b wave, lat	30.00	3.42	33.00	1.86						
Rr a wave, ampl	4.12	1.35	4.67	1.84						
Rr a wave, lat	13.31	1.30	14.14	1.63						
Rr b wave, ampl	6.73	2.64	8.51	1.95						
Rr b wave, lat	30.12	3.42	33.19	2.23						

### a. EOG

Depressed patients (as a whole), manifested a decrease of both dark trough and light peak values in comparison to controls. This did not hold true for Arden ratios or A-Criterion values which both were within normal range (table 1). This was true both for melancholic and atypical patients. The comparison between melancholic and atypical patients provided no significant results (table 2). However, both groups differed from controls.

Correlation analysis included only depressed patients. Both Arden ratios related negatively with the score in NDDS 1965, but this was significant only from the left eye (R = -0.48, p < 0.01). Left Arden ratio marginally correlated with the number of life events (R = 0.46), and the HDRS anxiety index (R = -0.47).

Concerning the existence of individual symptoms, according to DSM-IV and ICD-10 lists, patients with 'distinct quality of depressed mood' had lower right Arden ratio values (p < 0.001); patients who were 'worse in the morning' had lower right Arden ratio and right A-Criterion values (p < 0.001) and higher right dark trough values (p < 0.001).

### b. flash-ERG

Flash-ERG results suggested no differences between depressed patients and controls (table 1), nor between specific symptoms and controls exist (table 2).

There were correlations between b-wave latency and GAF (left eye, R = -0.55), number of atypical features (right eye, R = -0.50), number of life events (left eye, R = -0.49), non-specific HDRS index (bilaterally, R = 0.51).

There was also a positive correlation between HDRS depressed index and b-wave amplitude bilaterally (R = 0.52).

Concerning the existence of individual symptoms, according to DSM-IV and ICD-10 lists, patients with 'melancholic anhedonia' had bilaterally larger b- wave latency and those with 'thoughts of death' (present at the time of clinical interview) had prolonged b- wave latency (p < 0.001)

## Discussion

The alteration between light and dark produces electrochemical changes in the retina. The electro-oculogram (EOG) is a technique suitable for the study of the electrical and metabolic activity of the outer layers of the retina. The fall of a light stimuli on the retina produces early and late potentials. The method of recording late potentials is the Electroretinogram (ERG). ERG provides information about the functioning of the photoreceptors and the neuronal elements of the retina.

Both EOG and ERG in fact are useful indices reflecting dopamine activity. There are several studies in the international literature concerning the relationship of dopamine with specific depressive symptoms.

There is no report in the international literature on a combined use of EOG and ERG in depression. There are only papers using either method. This is one of the reasons the results and interpretations are inconclusive and problematic.

### a. EOG

The current study reports that although Arden ratios and A-criteria were within normal limits, both dark trough and light peak were reduced in all subtypes of depression. However, different mechanisms are reported to underlie them [31]. The light peak is related mostly to the intensity of the stimuli, while the dark through does not. Also, the light peak is related to the pre-adaptation level of the retina, while the dark trough is stable after only 2 minutes in dark. Generally, the standing potential of the eye manifests a diurnal rhythm, similar to that of the body temperature. It seems that after 15 minutes of adaptation to darkness, the amplitude of the dark trough is related only to the diurnal rhythm (in normal subjects).

The correlations between EOG variables and clinical picture and psychometric scales suggested that the core feature was the relationship of the dark trough with melancholic symptoms (NDDS 1965 score). Here again should be stressed that NDDS 1965 takes into consideration premorbid personality and personal history of affective illness, while the rest melancholic scales are largely cross-sectional and do not include personality assessment. Dark trough was of course lower than in controls, but this finding suggests that the more melancholic features the patient fulfilled, the closer its dark trough amplitude was to normal.

It is believed that the biochemical alterations, which produce the EOG potentials take place in the pigmentum epithelium. The origin of the light peak and dark trough probably lies in the interaction between photoreceptors and pigmentum epithelium [32], and dopamine seems to hold a major role [24,25,32-35]. The role of melatonin which is also reported to dysfunction in depression [12] remains elusive [36].

Thus, the EOG findings of the current study could receive two different interpretations: either dopamine activity is decreased, or an advance of the circadian cycle might be present, as already some authors have proposed [37,38]. Of course a combination of them could be present but this is not in accord with the results of the current study, since ERG findings were not significant. It is also possible that one of them could be the result of the other. Another important finding was the relationship of dark trough with melancholia.

There are no reports in the international literature concerning the different subtypes of depression. There are only a few papers, and focus on seasonal depression. Reports are inconclusive [39-45]. Light therapy acts on the photoreceptors, at least in the initial phase [46]. Lam [47] studied the EOG in 19 seasonal patients and reported the presence of subtle disorders in the retina, at the photoreceptors level, resulting in a decreased light sensitivity, evident from lower Arden ratios in depressed patients in comparison to controls. Terman et al [48] concluded that it is possible that some environmentally induced, but genetically determined state disorders of the photoreceptors contribute to the development of seasonal depression. They also suggested that these patients had light hypersensitivity due to cone hypereactivity. Beersma [49], suggested that this light hypersensitivity disturbs the information arriving to the hypothalamus via the retinohypothalamic tract (single neurone) and subsequently the functioning of the suprachiasmatic nucleus which seems to posses properties of an endogenous pacemaker which regulates the rhythms of the organism [50]. On the contrary, Reme [51] argues in favor of a reduced sensitivity to light in seasonal patients. The disturbed functioning [52] does not affect vision, but only those functions which demand prolonged exposure to light (similar to light therapy).

Leaving the area of seasonal depression, which is not the direct focus of the current study, two are the only papers investigating non-seasonal depression with EOG. Seggie et al [40] reported that there were no differences in the Arden ratios between 20 depressed patients and equal number of controls, however depressed patients had lower dark trough values. A careful study of the paper reveals that there was no similar finding concerning the light peak, probably due to small study sample, and if the study sample was larger, such a finding could be possible. The results of that study is to a large degree similar to ours. Seggie et al concluded that depressed patients were light supersensitive and located the disturbance at the receptor level, and specifically in the rods. The authors of the current study consider that these conclusions do not really fit the data of that study. Economou and Stefanis [39] studied unipolar and bipolar patients and reported lower Arden ratios in unipolar and higher in bipolars in comparison to controls. They concluded that the existence and the quality of psychomotor symptomatology and not the mood of the patients is of prime importance, and related their results to disorders of dopamine activity.

So, conclusively, in spite of the differences in interpretation, which is a difficult issue when only EOG is applied, the results reported in the international literature are in accord with the results of the current study.

### b. flash-ERG

The a- wave is produced in the photoreceptors as they respond to a light stimuli. The b-wave is neuronal in origin and largely reflects dopamine activity.

There are only scattered and unpublished reports (e.g. Seggie et al: Electroretinographic Changes in Depression, Proceedings of the 2^nd ^Canadian Workshop on Epiphysis, 1990), and all suggest that there is an increased amplitude and decreased latency of both the rods and the cones response to the flash-ERG. These findings support the existence of light hypersensitivity in depression. Similar observations were made in animals during the transition from light to dark conditions [53]. It has also been suggested that the retinal disorders might relate to a toxic effect of higher neurosteroid levels, which are produced on the basis of excitatory impulses from NMDA receptors through GABA_A _receptors. This arc is also influenced by the light of the environment [54].

The results of the current study do not confirm the finding of light hypersensitivity. Correlation results suggest that melancholic features related positively with the photoreceptor sensitivity in darkness, and this relationship seems to lie on a continuum.

### c. Synthesis of findings

The major theories related to our findings are [55]:

a. The phase advance hypothesis (Wehr and Wirz-Justice), which postulated that depressed patients get asleep too late in comparison to the rest of their rhythms.

b. The S deficiency hypothesis (Borbely and Wirz-Justice), which postulates that there is a disturbance in the homeostatic S procedure of sleep (reflecting the need of the organisation for sleep)

c. The adrenergic-cholinergic imbalance hypothesis of depression of Janowsky [56].

d. The proposal of von Zerssen et al [57] which suggests that rhythms are independent from depression and just intensify or attenuate the clinical picture in the same way they affect normal mood.

e. The internal coincidence theory, which basically focuses to the time of awakening. Wehr and Wirz-Justice again suggested that there is a 'depressiogenic switch' which normally is triggered and simultaneously inhibited by other synchronous activities; however in depressive patients the triggering occurs too early.

Wehr et al [58] tested the above theories by depriving 4 depressed patients (however only one unipolar) patients from the environment, and thus isolating the endogenous part of the rhythms. It is important to note that all patients were impressively eager to accept this deprivation and all were improved. They all expressed discomfort when the experiment ended. This last observation is of prime importance, since it can provide further data on the relationship between psychophysiological methods and abnormal but different response to light stimuli under different conditions, and stressful life events.

Another key report is that sleep deprivation, according to the review of Wu et al [59] immediately improves 67% of melancholic and 48% of neurotic depressives. If we combine this observation with the correlation of melancholia with the dark trough, one could conclude that higher dark trough values could predict better response to sleep deprivation. On the other hand, melancholics are considered not to respond well to light therapy and atypical (neurotic) patients share common clinical manifestations with seasonal depression.

Since all depressed patients (according to the results of the current study) had low dark trough and light peak values in comparison to controls, but normal ERG, it is most possible that the initial cause could lie in the pigmentum epithelium, which secondary could affect the functioning of the receptors. The change of rhythms could cause mild affective symptomatology in normal subjects [60], but in depression it is unlikely to be the prime disorder. Since lesions in the pigmentum epithelium have not been yet detected, this change in the functioning should be attributed to the change of the firing of the raphe nucleus, which is considered to be an endogenous pacemaker. There is no possibility of a spreading of the frontal lobe metabolism dysfunction seen in depression, to the retina, since, in the vast majority of cases, the ophthalmic artery stems from the internal carotid artery.

However, since no differences were evident between melancholic and atypical patients, the source of the difference in PR-VEPs latency between these two depressive subtypes [14] should be traced posterior to the retina and anterior to the chiasm. The problem is that the neurons that constitute the optic nerve have their body located in the ganglionic layer of the optic nerve, which constitutes the outer layer of the cerebral stratum, while their axons terminate in the lateral geniculate body. It is obvious that the part of the optic nerve from the retina to the chiasm constitutes only a part of the optic nerve axon, and thus it is very difficult to explain any dysfunction, which is so narrowly localized. The only thing that differentiates this specific area is the fact that its blood supply come from small vessels originating mainly from the anterior cerebral artery [61, 62 and 63]

There is another possibility. EOG, ERG and PR-VEPs are three different methods which can not be used simultaneously. Therefore, there might be some specific features (e.g. eye micromovements) which have different influence on each of these tests or are activated or deactivated during anyone of these tests, and thus contribute to the results reported. In this case, our effort to localize the dysfunction on the base of the results of our studies so far is in vain.

The advantages of the current study include the precise diagnosis according to modern diagnostic criteria and the detailed psychometric assessment. The major disadvantage is the deviations from the International Society for Clinical Electrophysiology of Vision (ISCEV) standards for the recordings of EOG and ERG.

## Conclusion

The main finding of the current study concerns the lower dark trough and light peak values while ERG findings were normal in all depressive subtypes. The above provide the empirical foundation in order to incorporate the reports of the international literature in a comprehensive theory, which could explain the mechanism of action of therapies like sleep deprivation and light therapy.

## Competing interests

The author(s) declare that they have no competing interests.
